# Pituitrin as an Ecbolic

**Published:** 1913-11

**Authors:** 


Pituitrin as an Ecbolic. M. T. Benson, Atlanta, *New Orleans M. and S. Jour.*, June, 1913. In seventy-seven cases, twenty-seven of which were primiparous, labor pains were promptly increased although forceps were applied in thirteen primiparous and six multiparous cases. Forty-four multiparæ were delivered in from twenty minutes to two hours.

